# Probing optically driven K_3_C_60_ thin films with an ultrafast voltmeter

**DOI:** 10.1063/4.0000295

**Published:** 2025-03-26

**Authors:** J. D. Adelinia, E. Wang, M. Chavez-Cervantes, T. Matsuyama, M. Fechner, M. Buzzi, G. Meier, A. Cavalleri

**Affiliations:** 1 Max Planck Institute for the Structure and Dynamics of Matter, Hamburg, Germany; 2 The Hamburg Centre for Ultrafast Imaging, Hamburg, Germany; 3 Department of Physics, Clarendon Laboratory, University of Oxford, Oxford, United Kingdom

## Abstract

Optically enhanced superconductivity in K_3_C_60_ is supported by transient optical spectra, by pressure responses, and by ultrafast nonlinear transport measurements. However, the underlying physics and in fact the similarity or dissimilarity to most properties of equilibrium superconductivity are not clear. In this paper, we study the ultrafast voltage response of optically driven K_3_C_60_ thin films. Photo-conductive switches are used to measure changes in voltage as a function of time after irradiation, both below and above T_c_. These measurements can be understood if one considers the role of granularity in the photo-induced transport response. They reveal fast voltage changes associated with the kinetic inductance of the in-grain carriers and a slower response that may be attributed to Josephson dynamics at the weak links. Fits to the data yield estimates of the in-grain photo-induced superfluid density after the drive and the dynamics of phase slips at the weak links. This work underscores the increasing ability to make electrical measurements at ultrafast speeds in optically driven quantum materials and demonstrates a striking new platform for optoelectronic device applications.

## INTRODUCTION

Optical excitation at mid-infrared and terahertz frequencies has emerged as a powerful technique to manipulate the properties of quantum materials,^1^ triggering intense research efforts aimed at achieving various types of non-equilibrium control of functional phenomena, such as magnetism,^2,3^ topological phases,^4,5^ and superconductivity.^6–16^

**FIG. 1.**
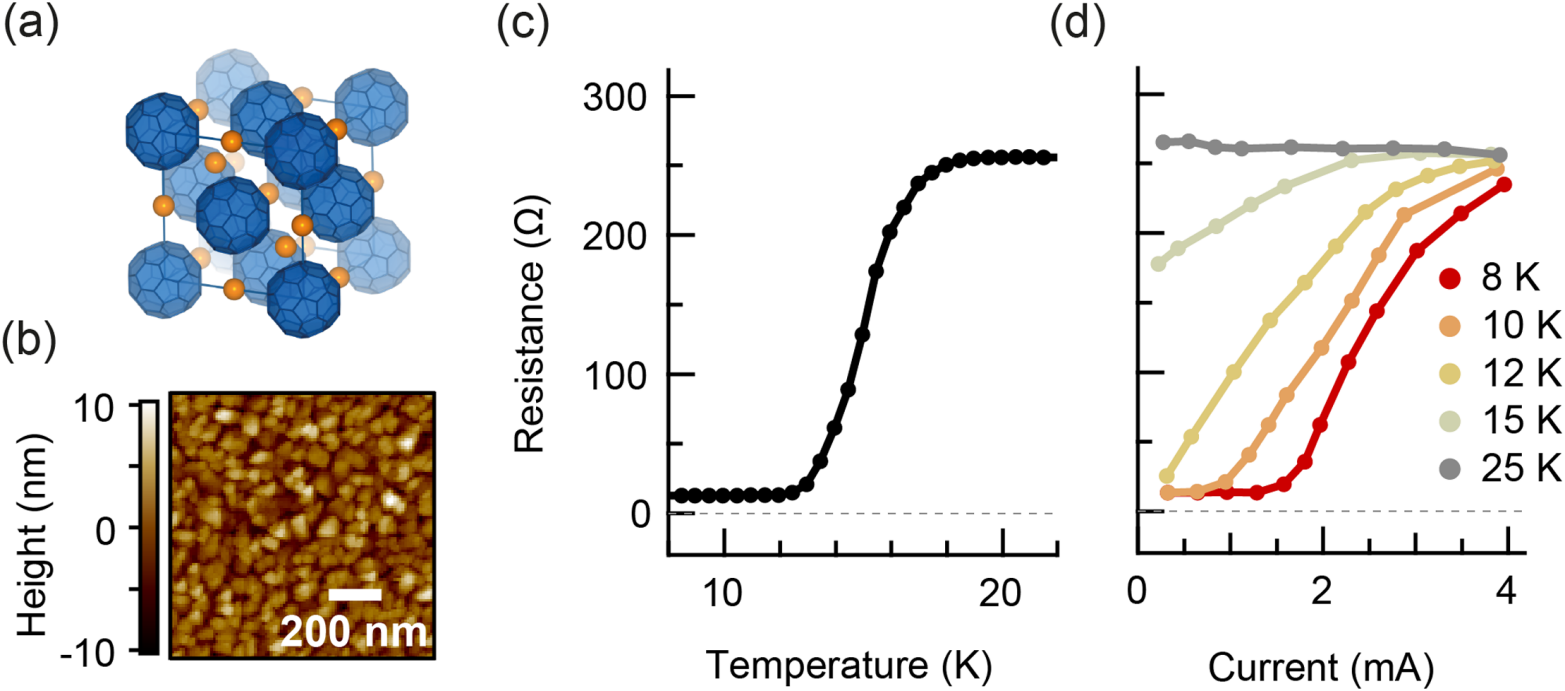
Crystal structure and equilibrium transport properties of thin-film K_3_C_60_. (a) Unit cell of K_3_C_60_.^17,18^ C_60_ molecules are represented in blue, and are situated at the lattice points of a face-centered cubic lattice. Potassium atoms are represented in orange. (b) Atomic force microscopy image of the MBE-grown C_60_ thin film before doping with Potassium. Grains of approximately 100 nm size are visible. (c) Two-contact DC measurement of resistance vs temperature of the K_3_C_60_ thin film. (d) Two-contact quasi-DC measurement of resistance vs current, for the temperatures 8, 10, 12, 15, and 25 K.

In the case of superconductivity, a number of experiments have focused on the alkali-doped fulleride K_3_C_60_ [Fig. 1(a)]. When excited with laser pulses in the mid^8–10^- and far^12^-infrared regime, K_3_C_60_ powder samples, held at base temperatures far above the equilibrium superconducting transition temperature T_c_, exhibited transient optical features that closely resembled those of the equilibrium superconducting state. Furthermore, a long-lived phase with vanishing electrical resistance has been reported in pressed K_3_C_60_ pellets,^10^ underscoring the similarities with an equilibrium superconductor.

These experiments have recently been complemented by a study in K_3_C_60_ thin films,^11^ which were grown for this purpose and integrated into an optoelectronic platform^5,11,19–22^ to study the transport properties with sub-picosecond current pulses. In those measurements, photo-excitation produced nonlinear current–voltage behavior at temperatures above T_c_, which was otherwise only present below T_c_ in equilibrium. The photo-excited transport properties above T_c_ bore a quantitative resemblance to those of the equilibrium state at 16 K—just below the transition temperature, where the granular constitution of the sample is understood to have a significant impact on superconducting transport.^23–25^ The response in these films was also found to be smaller and to survive up to a lower temperature than previously reported for powder samples.^8–10,12^

Here, we extend the capabilities of the optoelectronic platform developed in Wang *et al.*^11^ to measure the voltage dynamics across the K_3_C_60_ thin film upon photo-excitation in a quasi-DC bias. Such a configuration allows physical properties such as time-varying inductances and fast carrier dynamics to be accessed more directly than with the picosecond-long current pulses used in Ref. 11. We present a systematic study of photo-excited K_3_C_60_, reporting measurements for excitation at base temperatures below T_c_, where above-gap excitation is expected to disrupt superconductivity,^26,27^ and above T_c_, corresponding to the photo-induced superconducting-like features reported in previous works.^8–12^

Thin films of K_3_C_60_ were grown on sapphire substrates via molecular-beam epitaxy. An atomic force micrograph of the sample surface is shown in Fig. 1(b), illustrating a granular sample constitution with a grain size of approximately 100 nm. The resistance of the K_3_C_60_ thin film vs temperature was measured in DC in a two-contact configuration [Fig. 1(c)] and exhibited a broad superconducting transition with T_c_ = 19 K. The current dependence of the resistance, as measured under a quasi-DC bias (supplementary Sec. S2),^48^ is shown in Fig. 1(d). Nonlinear current–voltage characteristics, typical for superconductivity, were seen at temperatures below T_c_.

**FIG. 2.**
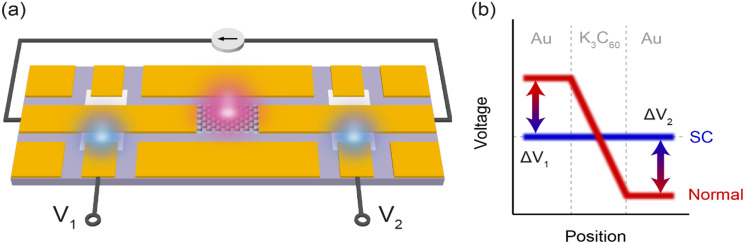
Ultrafast voltmeter. (a) Device architecture. An MBE-grown K_3_C_60_ thin film was incorporated into a coplanar waveguide, and biased with a quasi-DC. The K_3_C_60_ was then excited with a 300 fs duration, 7 *μ*m wavelength laser pulse at a fluence of 5 mJ/cm^2^ (indicated in red). The resulting perturbation to the voltage drop across the K_3_C_60_ thin film generated two electrical pulses which propagated left and right along the coplanar waveguide. The electrical pulses were detected on the left and right using silicon photo-conductive switches, which were operated with 250 fs gating pulses at 515 nm wavelength (indicated in cyan). (b) Sketch of the voltage across the device. The blue (red) line represents the voltage profile in the superconducting (normal) state of K_3_C_60_. The arrows represent the voltage changes at the positions of the photo-conductive switches during an ultrafast phase transition.

The device architecture for the ultrafast transport measurements is illustrated in Fig. 2(a). The K_3_C_60_ samples were connected to the signal line of a coplanar waveguide, which allowed for voltage transients to propagate at frequencies of up to 1 THz.^11,19,21^ The coplanar waveguide was contacted to photo-conductive switches^28^ on both sides of the sample, which were used to detect the transient voltages. The switches consisted of patches of amorphous silicon, which becomes conductive when excited with an ultrafast laser pulse in the visible spectrum.^28^ When photo-excited under a voltage bias, the switches launched current pulses with duration determined by the lifetime of the photo-excited carriers (< 1 ps) and a total charge proportional to the voltage bias, thus enabling sampling of transient voltages.^5^ By varying the time delay between the K_3_C_60_ excitation pulse (pump) and the switch trigger pulse (probe), a quantitative voltage measurement was obtained with sub-picosecond time resolution.

The K_3_C_60_ thin film was biased with a voltage pulse of 500 ns duration, long enough for the local current to stabilize (see the supplementary material, Fig. S2).^48^ After a constant current flow was established, the K_3_C_60_ was homogeneously excited with a mid-infrared laser pulse of 300 fs duration. In-current photo-excitation of the K_3_C_60_ caused changes in the voltage drop across the sample [Fig. 2(b)]. On the ultrafast timescales probed in the measurement, the voltage in the device was effectively floating, so changes in voltage across the K_3_C_60_ thin film resulted in equal and opposite voltage pulses propagating in the left and right directions. The voltage pulses were then detected at the photo-conductive switches as transient variations in the voltages V_1_ and V_2_. The switches were operated with laser pulses of 250 fs duration at 515 nm wavelength. The magnitude of the detected voltage changes was then calibrated using the procedure described in supplementary Sec. S3.^48^

## PHOTO-INDUCED VOLTAGE DYNAMICS BELOW T_C_

**FIG. 3.**
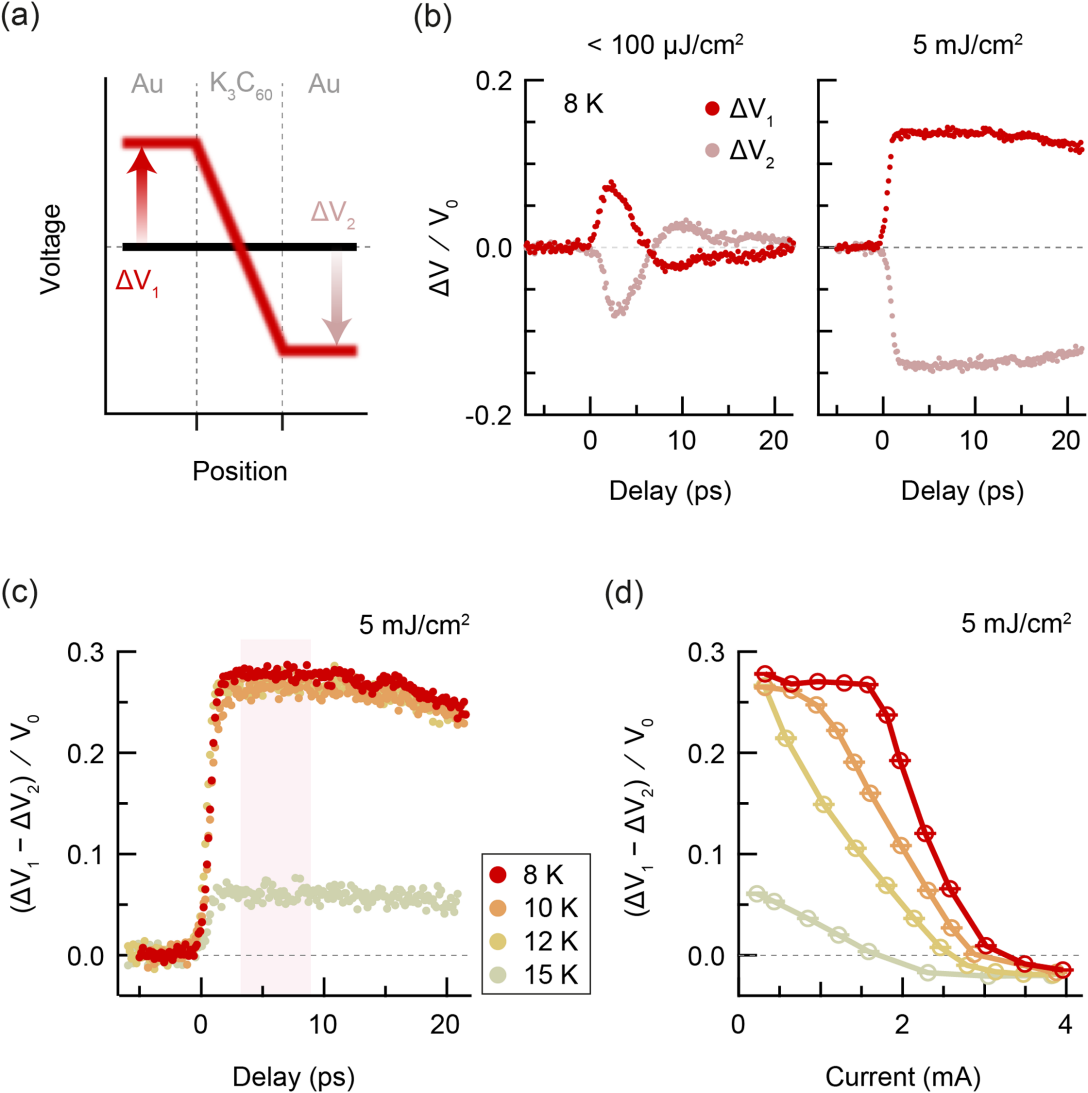
Photo-induced voltage dynamics in K_3_C_60_ (T < T_c_). (a) Sketch of the voltage across the device. The voltage profile changes from the equilibrium superconducting state (black) to the disrupted state (red) upon photo-excitation. (b) Pump-induced change in voltage on left and right of sample vs pump-gate delay, measured at 8 K, for a pump fluence of <100 µJ/cm^2^ (left) and 5 mJ/cm^2^ (right). (c) Pump-induced change in voltage drop across sample vs pump-gate delay, measured at 8, 10, 12, and 15 K, with an applied current of 0.3 mA. (d) Pump-induced change in voltage drop across sample vs applied current, measured at the same temperatures as in (c). Each data point is the mean value of the time-trace within a 6 ps window centered at 6 ps delay. The error bars represent the standard error of the mean change in voltage drop. All voltages are normalized by a factor V_0_, which is defined for the measurements below T_c_ as the applied current multiplied by the sample resistance at 20 K.

The first set of measurements was carried out at temperatures below T_c_. The photon energy of the excitation pulse was larger than the energy gap of the equilibrium superconducting state in K_3_C_60_.^29^ In this regime, mid-infrared excitation is expected to break Cooper pairs into unpaired electrons and disrupt superconductivity.^30^ Based on this, an increase in the voltage drop across the sample is expected^26,27^ [Fig. 3(a)].

The measured pump-induced changes in V_1_ and V_2_ (henceforth referred to as ΔV_1_ and ΔV_2_) are plotted in Fig. 3(b) as a function of pump-probe delay, at low (left) and high (right) excitation fluence. All voltages are normalized by a factor $V_{0}$, which, for the measurements below T_c_, is defined as the applied current multiplied by the sample resistance at 20 K. For low-fluence excitation, ΔV_1_ and ΔV_2_ had a bipolar shape. The positive peak in ΔV_1_ had a duration of 5 ps and was followed by a negative peak with a duration of 15 ps. For high-fluence excitation, ΔV_1_ exhibited a step-like increase with a rise time of approximately 1 ps. In all measurements, ΔV_2_ displayed equal and opposite behavior to ΔV_1_. For the given direction of applied current, an increase in ΔV_1_ indicates an increase in the total voltage drop across the sample, consistent with the disruption of superconductivity.

The total pump-induced change in voltage drop can be calculated as $\Delta V_{1}\;–\;\Delta V_{2}$. Figure 3(c) displays the pump-induced change in voltage drop across the K_3_C_60_ thin film for high-fluence excitation as a function of pump-probe delay for different temperatures below T_c_. Figure 3(d) displays the same normalized change in voltage drop as a function of applied current. Each data point in the current dependence is the mean value of the time-trace within a 6 ps window centered at 6 ps delay. At 8 K, the pump-probe signal was constant with the applied current up until 2 mA, above which the signal exhibited a step-like decrease. The step was consistent with the critical current of the equilibrium superconducting state [Fig. 1(d)]. Upon increasing temperature, the pump-probe signal began to decrease at lower currents and the step broadened.

## PHOTO-INDUCED VOLTAGE DYNAMICS ABOVE T_C_

**FIG. 4.**
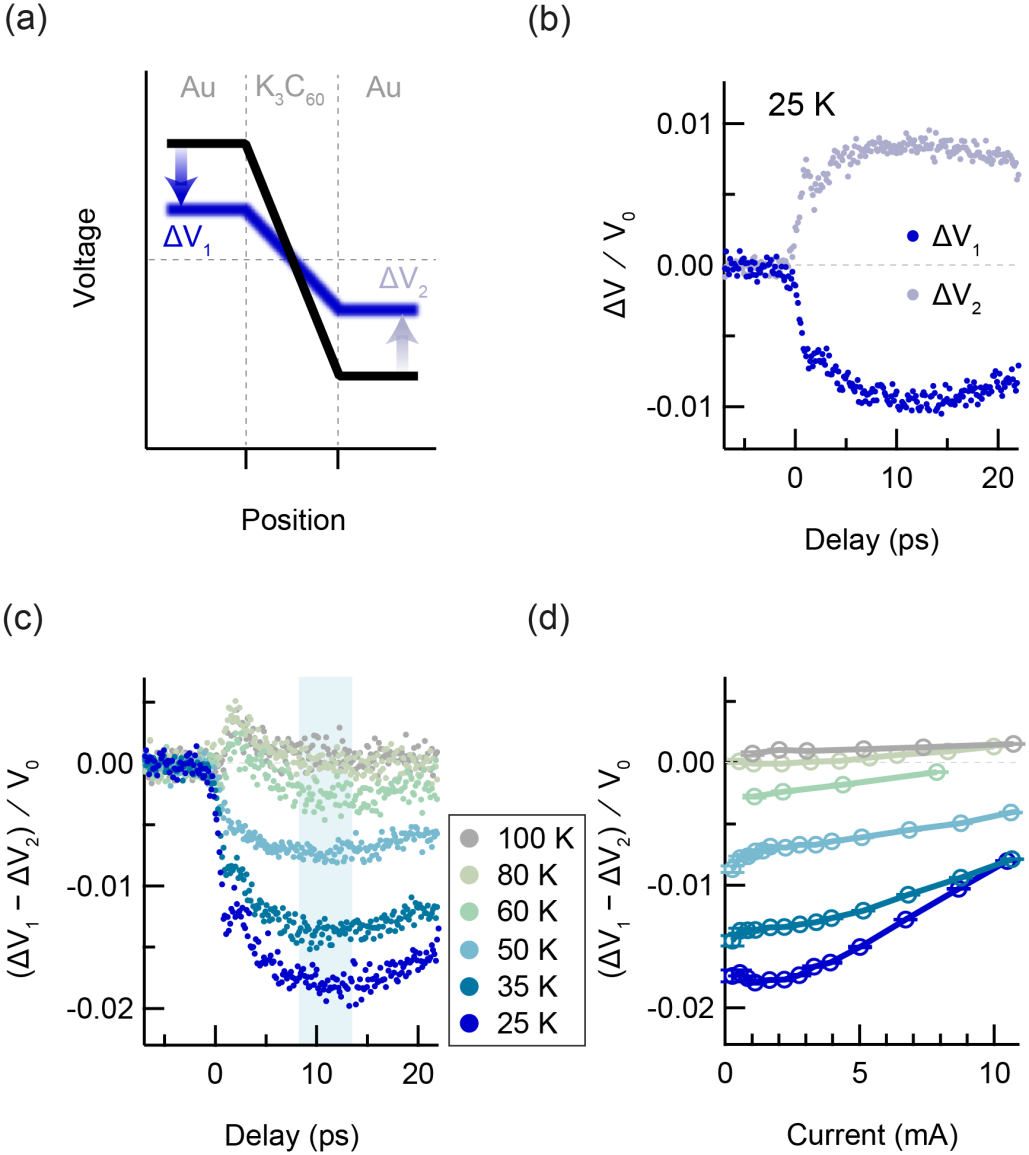
Photo-induced voltage dynamics in K_3_C_60_ (T > T_c_). (a) Sketch of the voltage across the device. The voltage profile changes when the sample is photo-excited from the equilibrium metallic state (black) into the nonequilibrium state (blue). (b) Pump-induced change in voltage on left and right of sample vs pump-gate delay, measured at 25 K for an excitation fluence of 5 mJ/cm^2^. (c) Pump-induced change in voltage drop across sample vs pump-gate delay, measured at 25, 35, 50, 60, 80, and 100 K, with an applied current of 1 mA. (d) Pump-induced change in voltage drop across sample vs applied current, measured at the same temperatures as in (c). Nonlinear I-V behavior is observable, which is also suppressed with increasing temperature. Each data point is the mean value of the respective time-trace within a 5 ps window centered at 11 ps delay. The error bars represent the standard error of the mean. All voltages are normalized by a factor V_0_, which is defined for the measurements above T_c_ as the applied current multiplied by the equilibrium sample resistance at the measurement temperature.

In a second set of measurements, we examined the photo-induced non-equilibrium state at temperatures above T_c_. The experimental setup was identical to the previous measurements below T_c_. Before photo-excitation, a voltage drop was present across the metallic K_3_C_60_ thin film corresponding to its equilibrium state resistance and the applied bias current. In this case, we expect photo-excitation at 7 µm wavelength to cause a decrease in voltage drop [Fig. 4(a)].

The measured ΔV_1_ and ΔV_2_ for photo-excitation at 25 K are plotted in Fig. 4(b) as a function of pump-probe delay. All voltages are normalized by a factor V_0_, which, for the measurements above T_c_, is defined as the applied bias multiplied by the sample resistance at the measurement temperature. The signs of ΔV_1_ and ΔV_2_ were reversed compared to the measurements below T_c_, indicating a reduction in voltage drop upon photo-excitation and an increase in the conductivity of the sample. ΔV_1_ became negative after photo-excitation, with dynamics present on two timescales; a fast, negative spike was present immediately after time-zero, which was followed by a slow decrease in ∼10 ps duration. ΔV_2_ displayed equal and opposite behavior to ΔV_1_. Figure 4(c) displays the pump-induced change in voltage drop across the K_3_C_60_ thin film as a function of pump-probe delay for different temperatures above T_c_. The magnitude of the voltage change decreased with increasing temperature until the sign of the response was reversed at 80 K. The positive response at high temperatures was also qualitatively different; only a single rise and decay was observed.

Figure 4(d) displays the voltage change as a function of the applied current. Each data point in the current dependence is the mean value of the time-trace within a 5 ps window centered at 11 ps delay. At 25 K, linear behavior in current was observed for small currents. Above 2 mA, the signal became nonlinear with the applied current. Upon increasing the temperature, the onset of the nonlinear behavior was shifted to lower currents. The nonlinear effect was also reduced alongside the signal magnitude at higher temperatures; the positive voltage change at 100 K was linear in current across the whole measurement range.

## DISCUSSION

We first turn our attention to the low-fluence excitation at 8 K [Fig. 3(b)], which displays bipolar, non-thermal voltage dynamics. Such a voltage response is consistent with changes in the kinetic inductance that are understood to originate from the breaking and subsequent recovery of Cooper pairs.^27,31–34^ The kinetic inductance $L_{K}$ of a conductor is given by^27^

$$L_{K}=\frac{\alpha }{n},$$ (1)

where α is a constant that depends on the sample geometry and the effective mass of the charge carriers and n is the charge carrier density. A change in the carrier density will therefore change the kinetic inductance. When this occurs in an applied current I, this generates an additional voltage

$$V_{K}=I\frac{dL_{K}}{dt}\propto -\frac{I}{n^{2}}\frac{dn}{dt}.$$ (2)

For a decrease in superfluid density, this voltage is positive. Similarly, the voltage becomes negative for a recovery of the superfluid density. We fit the voltage dynamics using a simple two-fluid model, consisting of a resistive channel in parallel with a purely inductive superconducting channel, under a variable superfluid density. All details of the model and fit are available in supplementary Sec. S4.^48^ For this weak excitation, Cooper pair-breaking and quasiparticle relaxation can be captured using a Rothwarf–Taylor time profile, followed by an exponential decay for the recovery of the superfluid.^35–37^ From the fits, we obtain time constants for Cooper pair-breaking $\left(\tau_{PB}=0.8\pm 0.1\;\text{ps}\right)$ and for the recovery of superconductivity $\left(\tau_{R}=50\pm 5\;\text{ps}\right)$.

In the high-fluence data below T_c_, the voltage shows a step-like increase with no recovery dynamics observed within our time window. The step-like increase results from the direct excitation of the Cooper pairs, with an estimated photon density ( $1.8\times 10^{17}$ cm^−2^) that is larger than the Cooper pair density ( $2\times 10^{16}$ cm^−2^).^38^ The plateau occurs because the pump pulse injects enough energy at this fluence to bring the sample temperature above T_c_. We also see that the temperature and current dependencies of the pump-probe signal directly coincide with changes in the equilibrium resistance shown in Fig. 1(d), so it is reasonable to conclude that the measured voltage changes at high fluence arise from a bolometric increase in the sample resistance.

**FIG. 5.**
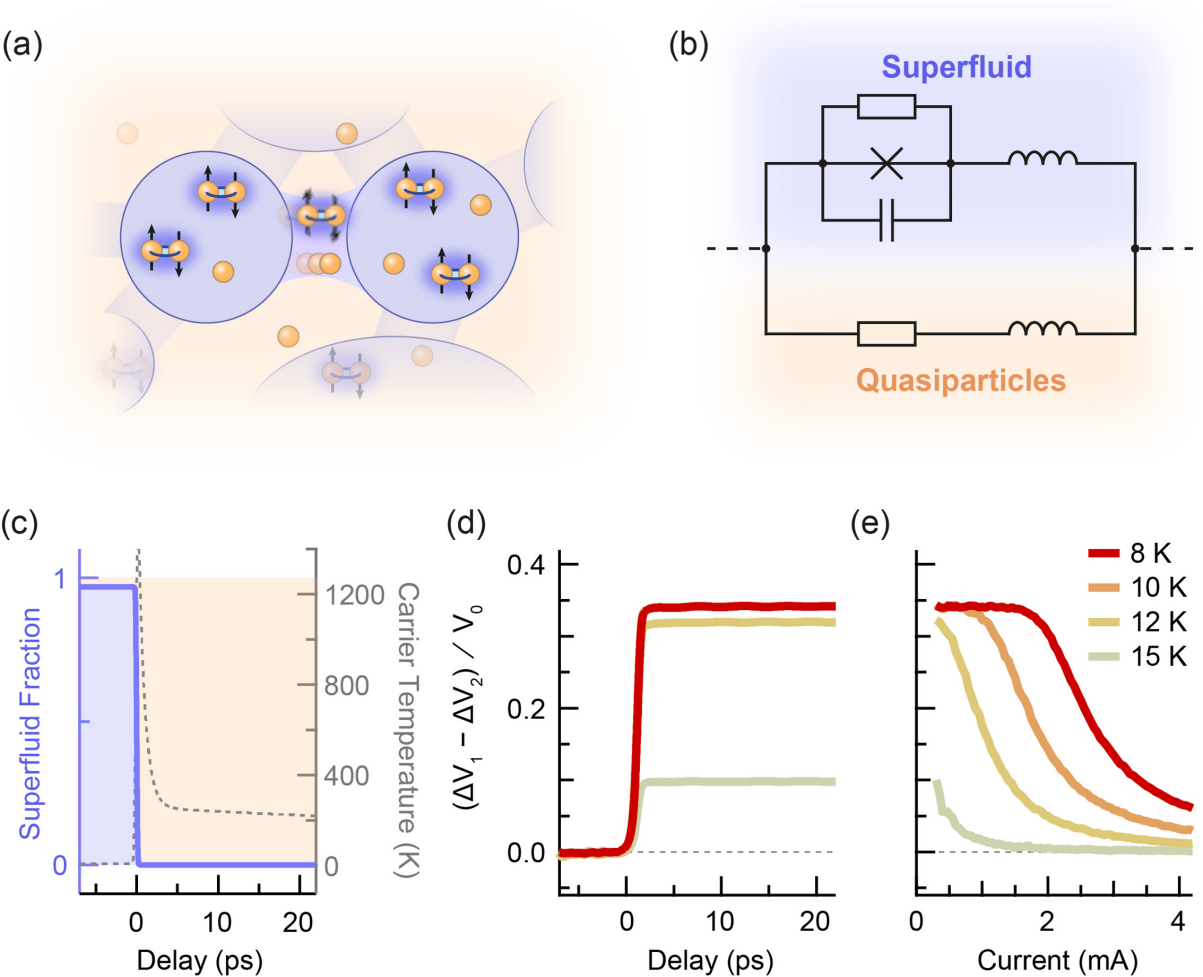
Disruption of superconductivity with a granular two-fluid model. (a) Sketch of a granular material with Cooper pairs and quasiparticles. Cooper pairs tunnel across grain boundaries, while thermal phase fluctuations disrupt phase coupling between the different superconducting grains. (b) Circuit diagram of the granular two-fluid model used to represent the K_3_C_60_ sample. The superfluid channel (highlighted in purple) consists of a typical RCSJ model extended to include the kinetic inductance of the superconducting grains. The resistive quasiparticle channel is highlighted in orange. (c) Time-dependent superfluid density used in the disruption simulation at 8 K. The superfluid fraction is assumed to reduce to zero at 0 ps delay. The carrier temperature (gray dashed line) was calculated using a two-temperature model. (d) Simulated change in voltage drop across sample vs delay, for base temperatures of 8, 10, 12, and 15 K, with an applied current of 0.3 mA. (e) Simulated change in voltage drop across sample vs applied current, for the same base temperatures as in (d). All voltages are normalized by a factor V_0_, which is defined for the measurements below T_c_ as the applied current multiplied by the sample resistance at 20 K.

The simple two-fluid model used to fit the data at low excitation fluence at 8 K is no longer adequate in the intermediate-temperature regime, since it produces a resistivity of zero for any finite superfluid density, and therefore cannot capture the finite resistivity in equilibrium for 12 K < T < 19 K. We now aim to construct a more sophisticated model that reproduces both the low-fluence and high-fluence measurements at all temperatures. Two factors should govern the physics of this model. First, disruption of superconductivity involves a transformation of charge carriers from Cooper pairs to resistive quasiparticles.^30^ The simple two-fluid model described previously therefore serves as a good foundation for the new model. Second, the granular constitution of the K_3_C_60_ thin film impedes dissipationless superconducting transport at temperatures just below the bulk T_c_, as evidenced in equilibrium by the broadened superconducting transition [Fig. 1(d)]. This is understood to result from thermally activated phase slips between grain boundaries and can be modeled using a resistively and capacitively shunted Josephson junction (RCSJ) model.^39^ To capture the sample properties before time-zero, we incorporate the RCSJ model into the superconducting channel of a two-fluid model, forming a granular two-fluid model, in order to simulate the voltage dynamics in a granular superconductor [Fig. 5(b)]. All details of the granular two-fluid model are available in supplementary Sec. S4.^48^

The voltage dynamics for the disruption of superconductivity under a DC bias were simulated by numerically solving the circuit equations for the granular two-fluid model under a changing superfluid density. The effect of temperature was modeled by considering thermal fluctuations in the phases of the order parameters of the superconducting grains, which result in thermally activated phase slips and the onset of resistivity at temperatures close to T_c_. We have assumed that, before time-zero, the superfluid density $n_{s}$ depends on temperature T following the relation:^40^

$$n_{s}\propto 1-{\left(T/T_{c}\right)}^{4}.$$ (3)

Here, we will focus on the simulation results for high-fluence excitation (the results for the low-fluence case are displayed in supplementary Fig. S23).^48^ At time-zero, the superfluid density was assumed to reduce from $n_{s}\left(T\right)$ to zero, as shown in Fig. 5(c), and the temperature increase upon excitation was calculated following a two-temperature model^33,34,41^ detailed in supplementary Sec. S4.2.^48^ The calculated voltage transients are displayed in Figs. 5(d) and 5(e) as functions of delay and current, respectively. The results from the granular two-fluid model exhibit strong agreement with the experimental results in Fig. 3, indicating that the voltage changes are consistent with a complete disruption of superconductivity for high-fluence excitation. In particular, both the near-T_c_ and high-current regimes are well-captured by the model, enabling analysis of carrier and thermal dynamics in inhomogeneous superconductors.

To interpret the experimental data above T_c_ (Fig. 4), we first consider the fast, negative voltage spike on short timescales. This fast response is notably not present when measuring the sample transmittance with picosecond current pulses,^11^ and this discrepancy is consistent with the fast response originating from a time-dependent kinetic inductance. Here, a negative V∝dL/dt arises from an increase or transfer of carrier density n. Notably, the negative voltage spike in the T > T_c_ data is significantly faster than the positive spike in the low-fluence, T < T_c_ data [Fig. 3(b)], which can be understood as the changes in n occurring on the timescale of the excitation pulse duration.

Since the quasi-DC is already present before photo-excitation, the nonlinear current voltage characteristics in Fig. 4(d) are somewhat ambiguous; they could be taken as a signature of a critical current in the photo-excited state, but could also arise from a suppression of the photo-susceptibility due to the current. At temperatures below T_c_, the transient voltage drop became negative at large DC currents [Fig. 3(d)]. In this regime, the equilibrium superconducting state was suppressed by the supercritical current before excitation. The optical drive therefore acted upon the metallic state, just like the above-T_c_ case, resulting in similar voltage dynamics (see supplementary Sec. S5).^48^

The photo-induced response exhibits a sign change at 80 K that can neither be explained by thermal effects, nor by a photocarrier response. This, together with the inductive response, nonlinear I-V characteristics,^11^ and optical conductivity features in the photo-excited state,^8–10,12^ suggests that the photo-excited transport response could be related to superconductivity in this material.

In an equilibrium superconductor, thermal phase fluctuations impede dissipationless transport across grain boundaries,^39^ and therefore, phase fluctuations must also play a role in the photo-excited state. We hypothesize that the slow voltage response on longer timescales results from the thermal dynamics of these phase fluctuations after photo-excitation. It is evident that high-fluence photo-excitation will increase the temperature of the system. The phase must therefore be highly disordered upon photo-excitation, and the initial resistivity of the photo-excited state at early delays would be left unchanged from that of the metallic state. As the phase thermalizes, the rate of thermally induced phase slips decreases, and the resistivity drops on a timescale of several picoseconds.^42,43^ Nevertheless, the phase slip rate at longer delays would be significant due to lattice heating, resulting in finite resistivity in the long-lived state after photo-excitation.

**FIG. 6.**
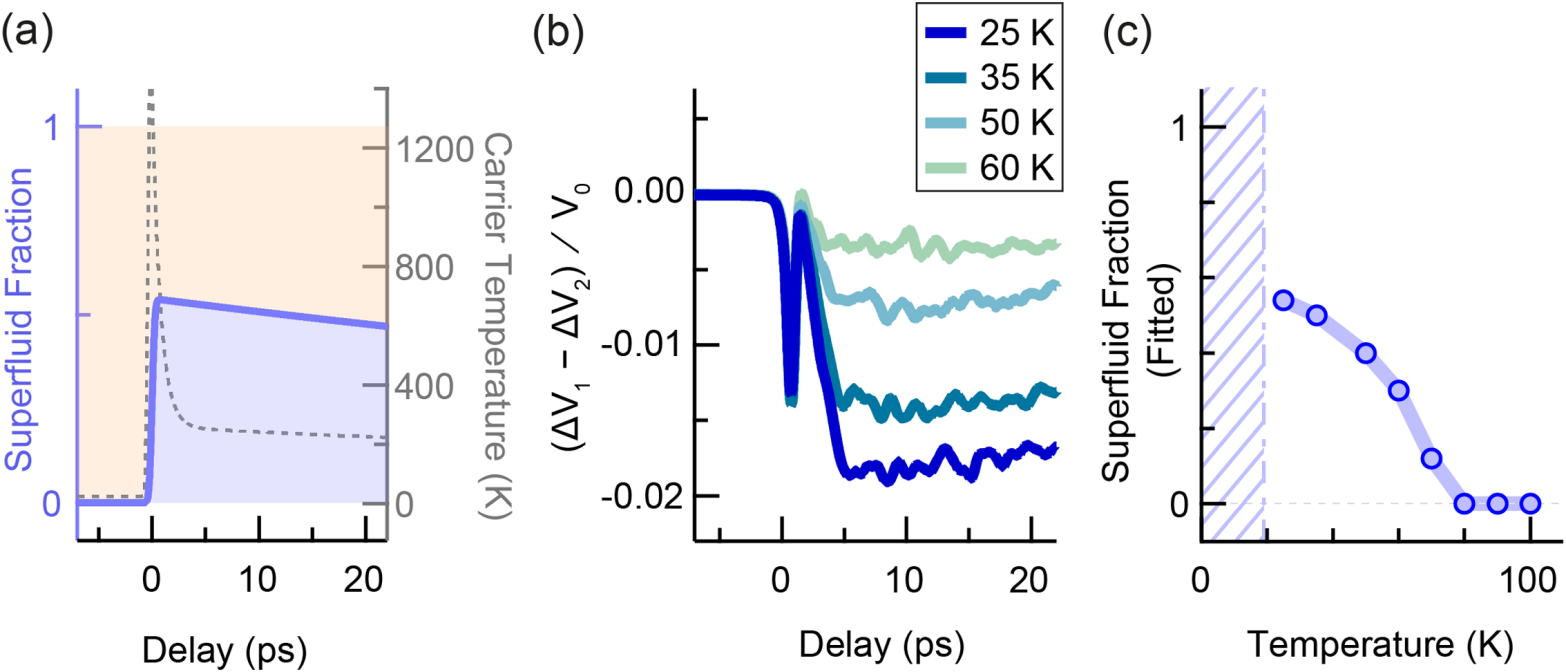
Photo-induced superconductivity with a granular two-fluid model. (a) Time profile of superfluid fraction for the simulation of the photo-excited state. The superfluid fraction (purple) is assumed to increase from zero to a finite value at 0 ps delay, and then to decay exponentially with a time constant of 150 ps. The carrier temperature (dashed gray line) was calculated using a two-temperature model. (b) Simulated change in voltage drop across sample vs delay, using peak superfluid fraction as the fitting parameter, for base temperatures of 25, 35, 50, and 60 K, with an applied current of 1 mA. All voltages are normalized by a factor V_0_, which is defined for the measurements above T_c_ as the applied current multiplied by the equilibrium sample resistance at the measurement temperature. (c) Light-induced “superfluid fraction” vs temperature as obtained from the fitting procedure described in the text. The blue symbols indicate granular two-fluid model fits to the voltage data on the granular thin films. The purple shaded region indicates temperatures below the equilibrium T_c_.

Both the inductive response and the effect of thermally induced phase fluctuations should be captured by the previously discussed granular two-fluid model. We use this model to simulate a fast onset of superconductivity, using the same circuit parameters as the below-T_c_ discussion, and display the results in Fig. 6. The superfluid fraction [Fig. 6(a), solid line] was assumed to increase from zero to a finite value at time-zero, with a rise time of 300 fs, and to subsequently decay exponentially in time with a time constant of 150 ps (see supplementary material of Ref. 11). The carrier temperature is estimated in Fig. 6(a) (dashed line) and results in a time-dependent rate of thermally induced phase slips in the photoexcited state. The circuit simulation at each temperature was used to fit the slow response in the data at 10 ps delay, using the peak superfluid fraction at time-zero as a fitting parameter. Other parameters, such as the rise and decay times for the superfluid fraction, were left unchanged between temperatures. The calculation of the time-dependent carrier temperature is available in supplementary Sec. S4.^48^

The fits are displayed in Fig. 6(b). The two-timescale dynamics seen in the measurement are also present in the circuit calculations, with a fast inductive response appearing at time-zero, followed by a slower dip in voltage. The superfluid fraction fitting parameter is plotted in Fig. 6(c) as a function of temperature and decreases with increasing temperature. For temperatures of 80 K and above, the experimental data no longer display a reduction in resistivity and are represented with a superfluid fraction of zero in place of a fit. These results demonstrate the competition between the nonlinear excitation and the inevitable injection of thermal energy into the system. Furthermore, this analysis framework allows us to quantifiably link the dynamics of microscopic phase fluctuations to the macroscopic transport properties.

In summary, we report ultrafast voltage measurements on K_3_C_60_ thin films upon in-current photo-excitation, and characterized the response of the system for both the photo-induced disruption of the equilibrium low-temperature superconductor, as well as the photo-excited regime above T_c_ in which features compatible with a photo-induced superconducting state have been observed. We report a reduction in voltage drop when exciting into the non-equilibrium state above T_c_, and a two-fluid model based on a granular superconductor was used to interpret these findings. The data display a change in the kinetic inductance upon photo-excitation, followed by a slow reduction in resistivity which is consistent with the electron-lattice thermalization timescale and could be attributed to thermalization of phase fluctuations. The new understanding extracted from these data complements that obtained for the case of cuprates,^6,7^ for charge transfer salts,^14,15^ and for K_3_C_60_ powders,^8–10,12^ for which the responses were compatible with optically induced bulk superconductivity. It also presents an opportunity to investigate phenomena intrinsic to granular superconducting systems^44–47^ in out-of-equilibrium conditions. The ultrafast voltmeter platform developed here can be extended to other non-equilibrium superconductors and is crucial to understanding inhomogeneities and quenched dynamics in these systems.

## METHODS

### Device preparation

The photo-conductive switches and coplanar waveguide were fabricated on a sapphire substrate using optical lithography with a bilayer photoresist, followed by electron-beam evaporation. For the switches, 200 nm of Si was deposited, and for the coplanar waveguide, 10 nm Ti/280 nm Au was deposited. For the C_60_ growth, a shadow mask was made from a sapphire disk with a 20 × 20 µm^2^ square hole cut into the center, which was aligned to the device using a micro-manipulator under an optical microscope. A 100 nm C_60_ thin film was then grown via molecular-beam epitaxy with a deposition rate of ∼1 nm/min and a device temperature of 200 °C. A similar shadow mask was then used to deposit 10 nm Ti/350 nm Au to contact the C_60_ to the signal line of the coplanar waveguide. The C_60_ thin film was then doped with potassium in the MBE chamber at a temperature of 200 °C. During the doping process, the resistance of the film was monitored, and doping was stopped when the resistance reached a minimum. After doping, the device was transferred into an Argon glovebox in a high-vacuum suitcase, where it was sealed with a diamond window, indium gasket, and Torr Seal vacuum epoxy. Additional details about the device fabrication process can be found in Wang *et al.*^11^

### Optical setup

The experiment was carried out using a Pharos laser with 1030 nm output wavelength at 50 kHz repetition rate. The mid-infrared laser pulses were generated using an optical parametric amplifier based on silver thiogallate and were chopped at a frequency of 1 kHz. The 515 nm pulses were generated using beta barium borate for second-harmonic generation of the fundamental. Both were focused onto the device through the cryostat window using a reflective objective.

### Electronic setup

The quasi-DC was driven by applying 500 ns voltage pulses to the signal line of the device. These pulses were generated externally with an Agilent 81150 A pulse generator, which was triggered at the 50 kHz laser repetition frequency. To measure the voltage transients, the output from each photo-conductive switch was sent into an electronic amplifier chain consisting of a custom-built transimpedance amplifier, followed by a lock-in amplifier which was triggered at the 1 kHz chopping frequency of the mid-infrared beam. Time-traces were obtained by scanning the time delay between the mid-infrared beam and the 515 nm beams using optical delay stages.

## Data Availability

The data that support the findings of this study are available from the corresponding authors upon reasonable request.
